# Research Note: It's not just stress**—**fecal contamination of plumage may affect feather corticosterone concentration

**DOI:** 10.1016/j.psj.2021.101494

**Published:** 2021-09-16

**Authors:** T. Bartels, J. Berk, K. Cramer, E. Kanitz, W. Otten

**Affiliations:** ⁎ Friedrich-Loeffler-Institut, Institute for Animal Welfare and Animal Husbandry, Celle, Germany; † University of Leipzig, Clinic for Birds and Reptiles, Leipzig, Germany; ‡ Research Institute for Farm Animal Biology (FBN), Institute of Behavioural Physiology, Dummerstorf, Germany

**Keywords:** chicken, stress indicator, corticosterone metabolite, feather, fecal contamination

## Abstract

The feather corticosterone concentration (**fCORT**) is increasingly used to assess long-term stress in birds as this indicator provides the potential to retrospectively evaluate the adrenocortical activity of a bird during the growth period of a feather over several weeks in one sample. However, there is still a lack of knowledge concerning external factors which can potentially influence fCORT in birds. The aim of the present study was to determine whether fCORT in laying hens is affected by previous fecal contamination of the plumage. Fully grown primaries 5 (**P5s**) of laying hens (n = 40) were used for the study. To test the effects of fecal contamination on fCORT, freshly defecated droppings from laying hens were collected and mixed manually. In order to simulate practical and at the same time standardized contamination, the upper surface of the previously determined middle third of the right P5 of each hen then was evenly coated with the paste prepared from fresh feces. The treated feathers were stored for 24 h protected from light at room temperature under a film cover to prevent evaporation. Thereafter, the applied layer of excreta was removed manually. Contralateral P5s of the same individuals were stored under identical conditions and served as controls. Both treatment and control feathers were washed in an aqueous soap solution, rinsed several times in pure water and air-dried subsequently. After pulverization and methanolic extraction, fCORT was analyzed by ELISA. The mean fCORT of treatment P5s (reference ‘feather length’: 12.88 ± 3.16 pg/mm; reference ‘feather weight’: 7.81 pg/mg ± 1.86 pg/mg) were significantly higher (*P* < 0.001) than those of control P5s (reference ‘feather length’: 9.76 ± 2.42 pg/mm; reference ‘feather weight’: 5.93 ± 1.44 pg/mg). Our results show that previous contamination with excreta can increase fCORT, which was detectable even after a washing procedure prior to analysis. In conclusion, fecal contamination of feathers is a significant influencing factor that has to be considered when applying fCORT measurements.

## INTRODUCTION

Minimally invasive sampling procedures for glucocorticoid measurement are becoming increasingly important in stress research in wild and captive animals as well as in domestic livestock (Palme, 2019). The feather corticosterone concentration (**fCORT**) is a promising marker of increased hypothalamic-pituitary-adrenal axis activity caused by repeated or long-term stress (Bortolotti et al., 2008). Thus, analysis of fCORT is used more widely to assess long-term stress in birds as it also provides the capability to retrospectively assess the adrenocortical activity during the growth period of a feather over several weeks in a single sample (Romero and Fairhurst, 2016). However, there is still a lack of knowledge regarding the mechanisms of corticosterone incorporation into the feather and also concerning the potential factors that influence fCORT in birds (Romero and Fairhurst, 2016). To date, only few studies have investigated the impact of external factors on fCORT, especially in relation to husbandry conditions in commercial poultry farming (Häffelin et al., 2020). According to Bortolotti et al. (2009) and Romero and Fairhurst (2016), fCORT appears to be stable over many decades and is resistant to heat exposure up to 75°C. Other external factors such as feather abrasion or sunlight and UV-irradiation may possibly affect fCORT, either due to physical damage of feather material, which may reduce the amount of fCORT by simple mass loss, or due to chemical degradation of corticosterone (Romero and Fairhurst, 2016). The latter may be a problematic factor in determining true fCORT accumulation, as such alterations are not visually detectable (Romero and Fairhurst, 2016). Häffelin et al. (2020) did not find any effects on fCORT after exposure of feathers to artificial UV-A radiation, but pointed out that the effect of UV radiation on fCORT remains to be studied in detail especially in poultry under free range conditions as natural UV radiation percentage varies during seasons, time of day, and location. In addition, a study of Aharon-Rotman et al. (2021) indicates that effects of external factors such as superficial coverings, for example, secretions of the uropygial gland, should be considered in the interpretation of fCORT values and need to be further investigated. Thus, before being able to use fCORT as a reliable indicator of stress, it is important to investigate external factors that can potentially decrease or increase fCORT.

In mammals, external factors and especially contamination with cortisol-containing urine, feces and saliva have been found to be important confounders of hair cortisol concentrations (Otten et al., 2020), whereas in birds, effects of external contaminations have not yet been studied. Farmed poultry are usually kept at high stocking densities, so that the birds frequently come into contact with excreta, both their own and that of their conspecifics. Both direct contact with droppings and indirect contact via soiled litter can result in varying degrees of plumage contamination. Droppings of chickens contain variable amounts of corticosterone and its metabolites (Rettenbacher et al., 2004; Möstl et al., 2005), and therefore contamination of plumage may cause incorporation and distorted fCORT values. The aim of the present study was to analyze whether fCORT in laying hens is affected by previous excremental plumage contamination, which might lead to an incorporation of corticosterone into the keratin matrix of the feather vane.

## ANIMALS, MATERIALS, AND METHODS

Husbandry conditions and all procedures performed in this study involving animal handling and treatments were in accordance with the German animal protection law. Approval by the local ethics committee was not required as all clipped feathers were fully grown, and no innervated and vascularized tissue was removed.

### Animals, Housing and Management

For the study, fully grown third-generation primaries 5 (**P5s**) of 40 brown-feathered laying hens (age: 134 d) of the New Hampshire line “L68” were used. Chickens were kept in a flock in one barn compartment (27 m²), littered down with wood shavings and equipped with perches (heights 70 cm). The compartments were climate controlled and shielded lightproof against external light. The lighting was provided at an intensity of at least 20 lx, and the lighting program was set to a 16L:8D light-dark cycle. The daily photoperiod was between 5:00 a.m. and 9:00 p.m. and included a 20-min twilight phase in the mornings and evenings. The laying hens were fed ad libitum with a homemade complete feed for layers. Drinking water was also provided unlimited.

### Experimental Design

In each individual, P5s of both wings were clipped with a wire cutter within the range of the feather quill beneath the superior umbilicus. Prior to further treatments, all feathers were checked for integrity, in particular for physical damage of the feather vane and the occurrence of fault bars. According to Bortolotti et al. (2008), only the vaned portion of the feather was used for further analyses. Since the distal part of the feather is exposed to increased mechanical stress and, accordingly, enhanced wear and alteration of this feather section is common, the central part of the feather vane was selected for experimental contamination. Hence, the length of each feather vane was determined and the vane was divided into 3 equal sections. Fresh droppings from laying hens were collected, cleaned from adhesions if necessary, and mixed manually. To simulate practical and at the same time standardized contamination, the upper surface of the middle section of the right P5 of each hen was then evenly coated with 5 g of the paste prepared from fresh feces without further addition of liquid. The treated feathers were stored protected from light at room temperature under a film cover as protection against evaporation. Contralateral P5s of the same individuals were stored under identical conditions and served as controls. For the treatment P5s, the applied layer of excreta was manually removed after an exposure time of 24 h. Feathers of both treatment and control group were washed by swirling and immersing in a lukewarm aqueous soap solution (1 %) for 30 s (Bortolotti et al., 2008). Subsequently, the feathers were rinsed twice for 30 s in ultrapure water and air-dried overnight. Thereafter, the P5s of both groups were stored protected from light at room temperature for 28 to 35 d until further processing.

### Analysis of Feather Corticosterone

Corticosterone extraction followed modified methods previously described by Bortolotti et al. (2008). After removing the calamus, each feather was coarsely chopped with scissors, frozen in liquid nitrogen and pulverized using a ball mill. Approximately 50 mg of the feather powder was mixed with 5 mL methanol, placed in an ultrasonic water bath at room temperature for 30 min and then incubated for 18 to 24 h. Feather corticosterone concentrations were analyzed in duplicate using a species-specific ELISA assay for corticosterone quantification in chicken (CSB-E11991C, CUSABIO, Wuhan, China). The detailed method for extraction and analysis of feather corticosterone used in the present study as well as information about analytical validation of the assay (precision, accuracy, specificity, and sensitivity) are described in Bartels et al. (2021). All measured values were related to feather length and feather mass, and corticosterone concentrations were presented in pg/mm and pg/mg. In addition, corticosterone concentrations of chicken dropping samples (n = 8; average mass: 3.57 g [SD 0.72 g]) were analyzed in duplicate after extraction with methanol (60%) using the same ELISA assay.

### Statistical Analysis

Statistical analyses were performed using the software SPSS Statistics (version 25; IBM, Armonk, NY). Data distribution was assessed for normality by the Shapiro-Wilk test. Significant differences between the 2 groups were tested by paired *t* tests. Values of *P* ≤ 0.05 were considered to be significant.

## RESULTS AND DISCUSSION

**Figure 1:**
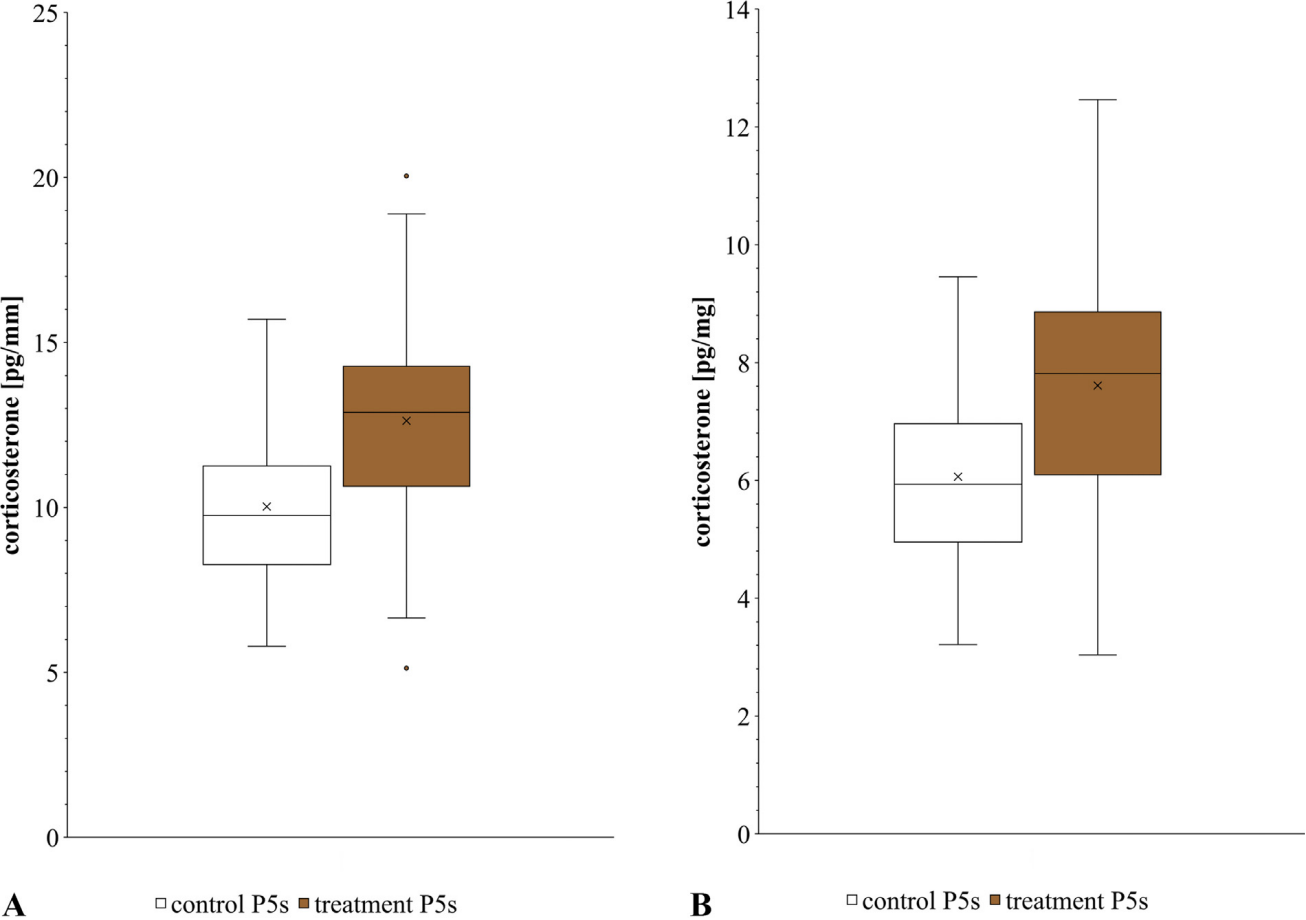
Feather corticosterone concentration of untreated and excreta-soiled primaries 5 of brown-feathered laying hens (n = 40) of the New Hampshire line “L 68” in relation to feather length (A) and feather mass (B). The box and whisker plots show the minimum value, 1st quartile, median, 3rd quartile and maximum value of a data set. Box: 1st to 3rd quartile; middle line of the box: median; x: mean; whiskers: variability outside the 1st and 3rd quartiles; outlier: value that lies more than 1.5 times outside the box. Differences between fCORT of treatment group and control group were significant (*P* < 0.001).

Our results show that mean corticosterone concentrations of treatment P5s (reference ‘feather length’: 12.88 pg/mm [SD 3.16 pg/mm]; reference ‘feather weight’: 7.81 pg/mg [SD 1.86 pg/mg]) were significantly higher (p < 0.001) than those of control P5s (reference ‘feather length’: 9.76 pg/mm [SD 2.42 pg/mm]; reference ‘feather weight’: 5.93 pg/mg [SD 1.44 pg/mg]) (Figures 1A, 1B). The fCORT of control feathers were within a comparable range as already found in feathers using the same ELISA assay in a previous study in laying hens (Bartels et al., 2021).

To the best of our knowledge, this is the first study to describe the potential effects of plumage contamination with excreta on fCORT. Prolonged contact of feathers with droppings of the same bird species, as it may occur daily under real conditions in commercial poultry farming, can lead to a significant increase in fCORT. Because both treatment and control feathers were obtained from the same individuals, feather length (148.78 mm [SD 4.01 mm]) and feather mass (247.72 mg [SD 23.67 mg]) of treatment P5s did not differ significantly from those of control P5s (feather length: 148.78 mm [SD 5.02 mm]; feather mass: 247.16 mg [SD 25.01 mg]). This ensured that manual contamination with feces resulted in an equal amount of excreta applied per length/weight unit of the clipped primaries. In the fecal samples used for fecal corticosterone extraction, we were able to detect mean fecal corticosterone concentrations of 7.01 pg/mg [SD 4.85 pg/mg] with the same assay. There is inconsistent information in the literature regarding the extent to which corticosterone is excreted with droppings in birds. Hepatic metabolization of corticosterone leads to the synthesis of various complex corticosterone metabolites that are excreted via the kidneys or the biliary tract (Möstl et al., 2005). According to Rettenbacher et al. (2004) corticosterone is extensively metabolized in droppings of healthy chickens, whereas Dehnhard et al. (2003) found an immunoreactive peak consistent with corticosterone in the HPLC immunogram of chicken feces. According to the manufacturer of our assay, the antibody used has high specificity for the detection of chicken corticosterone, however, further information on cross-reactivities of the antibody with other steroids was not provided. In the study of Dehnhard et al. (2003), an assay based on an antibody against corticosterone was proven as a valuable tool for the determination of fecal glucocorticoid metabolites in chicken (Dehnhard et al., 2003), indicating that cross-reactivities with other metabolites may exist.

The fully grown feather is a nonliving tissue, and therefore any addition of corticosterone into the feather once it is mature must be externally. It can thus be assumed that corticosterone and/or metabolites in feces were incorporated into the keratin matrix of the feather. Despite cleaning the treatment feathers in an aqueous soap solution, significantly increased glucocorticoid concentrations were found in feathers previously soiled with excreta. This finding confirms data from Bortolotti et al. (2008) who noticed that superficial cleaning of feathers with detergent solution did not decrease fCORT. Primaries of the same position in both left and right wing grow at the same time, thus are exposed to the same plasma corticosterone concentrations during feather growth and consequently do not differ significantly in fCORT (Häffelin et al., 2021). Intraindividual differences in fCORT deposition during feather growth can therefore not account for the fCORT differences between treatment and control feathers demonstrated in the present study. To date, there is a lack of knowledge about the mechanism by which corticosterone and metabolites enter the feather from excreta deposited on the feather. Presumably, the lipophilic properties of corticosterone and the specifics of feather keratinization play a crucial role. In addition to secretions from the uropygial gland, the presence of endogenous lipids within a feather and presumably also on the feather surface is of major importance for the hydrophobic and lipophilic properties of feathers (Zeisler-Diehl et al., 2020). This property also suggests the possibility of penetration of lipophilic substances from deposits on the feather surface. This could explain an incorporation of corticosterone and metabolites from excreta deposited on the feather into the feather material. However, this assumption still needs to be verified by further investigations, for example, by degreasing the feathers using solvents with highly effective lipophilic properties such as xylene prior to contamination of the feathers with excreta.

In conclusion, our results indicate that especially in poultry kept at high stocking densities, fecal contamination of plumage has to be considered as a factor with a potential impact on corticosterone concentration in feathers. However, further studies should investigate the influence of natural excreta contamination on fCORT under real housing conditions in laying hens and broiler chickens, as well as in other poultry species.
